# Thyroid Dysfunction at Different Stages of Chronic Kidney Disease: A Cross-Sectional Study at a Rural Teaching College in Central India

**DOI:** 10.7759/cureus.42130

**Published:** 2023-07-19

**Authors:** Ifthekar Ansari, Sunil Kumar, Sourya Acharya, Sachin Agrawal, Keyur Saboo

**Affiliations:** 1 Department of Medicine, Jawaharlal Nehru Medical College, Wardha, IND; 2 Department of Medicine, Datta Meghe Institute of Medical Sciences, Wardha, IND; 3 Department of Internal Medicine, Jawaharlal Nehru Medical College, Wardha, IND

**Keywords:** endocrine, thyroid stimulating hormone, low t4 syndrome, low t3 syndrome, chronic kidney disease

## Abstract

Background

The high prevalence of thyroid dysfunction in patients with chronic kidney disease (CKD) indicates a major correlation between the progression of CKD and thyroid dysfunction. In this study, we highlighted thyroid dysfunction and its relation to the severity and different stages of CKDs.

Materials and methods

From October 2018 to September 2020, 200 cases with CKD, admitted under the Department of Medicine at a rural teaching hospital in central India, were selected for the study. The collected data was analyzed and correlated using the Chi-square test, and the parameters suggested the presence or absence of low T3 syndrome, low T4 syndrome, and primary hypothyroidism.

Results

Out of 200 patients enrolled, 181 (91.5%) had thyroid abnormalities. Among these patients, the presence of low T3 syndrome was 57%, low T4 syndrome was 23%, and primary hypothyroidism was 10.5%. It was reported that as the CKD stages advanced, TSH levels increased with a statistically significant difference (p=0.04).

Conclusions

As kidney function progressively deteriorated, specifically in stage five, the chances of occurrence of hypothyroidism increased.

## Introduction

Previous reports have shown the prevalence of chronic kidney disease (CKD) ranging from <1% to 13%, and currently, statistics from the International Society of Nephrology's Kidney Disease Data Center have observed a prevalence of 17%. There is also a high prevalence of thyroid dysfunction in CKD patients [1,2]. The thyroid gland primarily secretes thyroxine (T4), which is peripherally converted to the more active form of tri-iodothyronine (T3). Local deiodination of T4 in the kidney by the isoform D1 of the enzyme T4-5′-deiodinase leads to the production of the T3 hormone [3]. As CKD progresses, the production of T4-5′-deiodinase decreases, leading to low T3 syndrome and clinical and subclinical hypothyroidism [4-6]. There are multiple factors responsible for thyroid disorders seen in CKD patients. The hypothalamus-pituitary-thyroid axis and the peripheral metabolism of thyroid hormone are affected in CKD. Low T3 and subclinical hypothyroidism is the most common thyroid disorder found in these patients. TSH levels are usually normal, as pituitary receptor response to TRH is blunted, causing a decrease in TSH release. The response of TSH to TRH is delayed because of the decreased clearance and the increase in the half-life of TSH [7,8]. The thyroid hormone affects the kidney through local and systemic hemodynamic changes and direct local effects. The thyroid hormone affects the glomerular filtration rate (GFR), proteinuria, and tubular function [9,10]. In CKD, there is reduced clearance of inflammatory cytokines such as TNF-alpha and IL-1. This leads to decreased peripheral conversion of T4 to T3, again leading to low T3 levels. This is because inflammatory cytokines lead to a decrease in the level of 1 5'-deiodinase, which converts T4 to T3 [11,12,13]. Hypothyroidism leads to decreased cardiac output due to negative chronotropic and inotropic effects, reducing renal blood flow [14]. Hypothyroidism also leads to an increase in peripheral vascular resistance [15], vasoconstriction in the renal vessels [16], a decrease in the renal response to vasodilators, and a decrease in the expression of renal vasodilators [17] such as insulin-like growth factor-1 (IGF-1) and vascular endothelial growth factor (VEGF) [18]. Another proposed mechanism is that there are changes in the glomerular structure, like basement membrane thickening and increased expression of the mesangial matrix. This leads to a decrease in renal blood flow [19]. Due to numerous reasons, in more than 55% of adults with hypothyroidism, the GFR is reversibly reduced by about 40% [20]. There is a reduced response to β-adrenergic stimulus and decreased renin release, with reduced angiotensin II and impaired renin-angiotensin-aldosterone system (RAAS) activity, resulting in the fall of GFR [21]. Hypothyroidism leads to a different effect on the kidney than hyperthyroidism [22-26]. Data on thyroid profiles in patients with CKD in the central part of India is scarce. Hence the present study was conducted to find out thyroid hormone dysfunction at different stages of CKD by estimating T3, T4, and TSH.

## Materials and methods

This cross-sectional observational study was conducted in the Department of Medicine at a rural teaching hospital in Wardha district in central India, from October 2018 to September 2020. This study was approved by the Institutional Ethical Committee Board at the Datta Meghe Institute of Medical Science [(DU)/IEC/2018-19/7557]. Two hundred patients with CKD, aged over 18 years, were enrolled in this study. The GFR was calculated using the Cockcroft-Gault formula, and the KDIGO guidelines were used for staging CKD. CKD was defined according to the KDIGO guidelines, which include: kidney damage for three or more months as defined by structural or functional abnormalities of the kidney, with or without decreased GFR. This can be manifest by pathological abnormalities, markers of kidney damage (including abnormalities in the composition of blood or urine or abnormalities in imaging tests), or eGFR less than 60ml/min/1.73m2 for three or more months, with or without kidney damage. Exclusion criteria involve patients with ongoing hemodialysis or peritoneal dialysis, proteinuria, diabetes mellitus, or hepatic disorder; drugs interfering with the thyroid levels like amiodarone, steroids, dopamine, phenytoin, beta-blockers, estrogen pills, iodine‑containing drugs; and chronic thyroid disorder history in the past. Before enrolling, the patient's detailed history and informed written consent were taken, including a general examination. Investigations for triiodothyronine (FT3), free thyroxine (FT4), and thyroid-stimulating hormone (TSH) were conducted using the electrochemiluminescence technique and kits from the Advia Centaur XP analyzer system of Siemens (Siemens Healthier Limited, Siemens Healthcare GmbH, Erlangen, Germany). The results from the participants were then analyzed. The primary outcome was to correlate the thyroid hormone dysfunction with different stages of CKD.

Statistical analysis

Collected data were analyzed using the Chi-square test. Descriptive analysis was done to study the presence or absence of low T3 syndrome, low T4 syndrome, primary hypothyroidism, and CKD.

## Results

The study group comprised 200 cases of CKD, with data on various demographic details, clinical characteristics, and thyroid profiles collected and subjected to statistical analysis. Of the 200 patients included in the study, 147 males and 53 females met the CKD criteria. All baseline characteristics are shown in Table 1. Table 2 shows the association of T3, T4, and TSH with the CKD stage. As CKD progresses, statistically significant decreases in the levels of T3 and T4 are observed (p=0.007 and p=0.01, respectively). The lowest levels of T3 and T4 were most commonly found in subjects at CKD stage 5, followed by stage 4. With the advance in CKD stages, TSH levels increased, a difference that was statistically significant (p=0.04). High TSH level was found more among subjects with CKD stage 5, followed by stage 4.

**Table 1:** Baseline characteristics of the study subjects. CKD: Chronic kidney disease; T3: Triiodothyronine; T4: Thyroxine.

Parameter		N=200	Percentage
Gender	Male	147	73.5
	Female	53	26.5
Age (years)	<30	15	7.5
	30-60	126	63
	>60	59	29.5
Stage of CKD	Stage 1	3	1.5
	Stage 2	5	2.5
	Stage 3a	6	3.0
	Stage 3b	10	5.0
	Stage 4	45	22.5
	Stage 5	131	65.5
Thyroid disorder	Euthyroidism	19	9.5
	Low T3 Syndrome	114	57.0
	Low T4 Syndrome	46	23.0
	Primary Hypothyroidism	21	10.5

**Table 2:** Association of T3, T4 and TSH with CKD stages. CKD: Chronic kidney disease; TSH: Thyroid stimulating hormone; T3: Triiodothyronine; T4: Thyroxine.

CKD Stage	T3		T4		TSH	
	Low	Normal	Low	Normal	High	Normal
	N (%)	N (%)	N (%)	N (%)	N (%)	N (%)
Stage 1	1 (0.7%)	2 (3.1%)	3 (4.5%)	0 (0.0%)	1 (4.8%)	2 (1.1%)
Stage 2	3 (2.2%)	2 (3.1%)	2 (3.0%)	3 (2.3%)	1 (4.8%)	4 (2.2%)
Stage 3a	5 (3.7%)	1 (1.5%)	2 (3.0%)	4 (3.0%)	1 (4.8%)	5 (2.8%)
Stage 3b	7 (5.2%)	3 (4.6%)	5 (7.5%)	5 (3.8%)	2 (9.5%)	8 (4.5%)
Stage 4	28 (20.7%)	17 (26.2%)	16 (23.9%)	29 (21.8%)	4 (19.0%)	41 (22.9%)
Stage 5	91 (67.4%)	40 (61.5%)	39 (58.2%)	92 (69.2%)	12 (57.1%)	119 (66.5%)
Total	135 (100.0%)	65 (100.0%)	67 (100.0%)	133 (100.0%)	21 (100.0%)	179 (100.0%)
P-value	0.02*	0.007*	0.02*	0.01*	0.04*	0.04*

Similarly, as the CKD stages advanced, the fT3 level decreased with a statistically significant difference (p=0.007). Low fT3 level was found more among subjects with CKD stage 5, followed by stage 4. Low fT4 level was found more among subjects with CKD stage 5, followed by stage 4 with a statistically significant difference (p=0.01). It was observed that as the CKD stages advances, the TSH level increases with a statistically significant difference (p=0.04). High TSH level was found more among subjects with CKD stage 5 followed by stage 4. All thyroid disorders, i.e., euthyroidism, low T3 syndrome, low T4 syndrome, and primary hypothyroidism, were reported most frequently among subjects with stage 5 CKD, followed by stage 4, with statistically significant differences, as shown in Table 3.

**Table 3:** Association of CKD stages with thyroid disorder. CKD: Chronic kidney disease; T3: Triiodothyronine; T4: Thyroxine.

Stages of CKD		Euthyroidism	Low T3 Syndrome	Low T4 Syndrome	Primary Hypothyroidism
Stage 5	N	13	78	27	13
	%	68.4%	68.4%	58.7%	61.9%
Stage 4	N	5	24	13	3
	%	26.3%	21.1%	28.3%	14.3%
Stage 3 b	N	0	5	3	2
	%	0.0%	4.4%	6.5%	9.5%
Stage 3 a	N	0	5	0	1
	%	0.0%	4.4%	0.0%	4.8%
Stage 2	N	1	2	1	1
	%	5.3%	1.8%	2.2%	4.8%
Stage 1	N	0	0	2	1
	%	0.0%	0.0%	4.3%	4.8%
Chi Square		9.19	8.13	7.41	7.82
P-value		0.02*	0.03*	0.04*	0.04*

## Discussion

In our study, it was observed that as the CKD stages advanced, the T3 levels decreased with a statistically significant difference (p=0.007). A study by Pan B et al. revealed that FT3 or T3 became more prevalent with increasing eGFR, with the lowest level in CKD stage 5 (p < 0.01). No significant differences were found between groups in FT4, T4, or TSH (p > 0.05) [27]. However, they did not find any correlation with other stages of CKD, as observed in our study. Low T3 levels were found more among subjects with CKD stage 5 followed by stage 4. It was also observed that as the CKD stages advanced, the T4 level decreased with a statistically significant difference (p=0.01). Low T4 levels were found more among subjects with CKD stage 5 followed by stage 4. This study has also revealed that as the CKD stages advanced, TSH levels increased with a statistically significant difference (p=0.04). High TSH level was found more among subjects with CKD stage 5, followed by stage 4. All thyroid disorders, i.e., low T3 syndrome, low T4 syndrome, and primary hypothyroidism, were reported most frequently among subjects with stage 5 CKD, followed by stage 4, with statistically significant differences. In our study, the level of T3 was found to be in a decreasing trend with increasing age. As age advances, there is reduced deiodination of T4 to form T3, leading to a decline in the T3 levels. Furthermore, the levels of anti-thyroperoxidase and anti-thyroglobulin antibodies rise with age, commonly seen in women above 60 years of age, contributing to the decline in the level of T3. In a study by Swaminathan K et al. [28], there were 50 patients included in the study, out of which nine patients were aged below 30 years, 35 patients were in between 31 and 60 years, and six patients were above 60 years of age, which was very indistinguishable from our study. In the present study, high TSH values were seen in 10.5% of the subjects, and low TSH levels in 89.5%. Low T3 was seen in 62.5% of subjects, and 33.5% of them had low T4. Out of 135 subjects with low T3 syndrome, 21 and 114 had high and normal TSH levels, respectively. Out of 67 subjects with low T4 syndrome, 21 and 46 had high and normal TSH levels, respectively. This study was well correlated with a similar study conducted by Chandra A [29-30]. Swaminathan K et al., in their study, found that 66%, that is, about 33 subjects had low serum T3 [28]. Primary hypothyroidism was found in four patients (8%) with low serum T3, low T4, and high TSH. It was found that, except for these four patients, 29 patients had low T3 syndrome, and 16 patients had low T4 levels. Excluding hypothyroidism, reduced T4 was found in 12 (24%) patients. The level of TSH in their study varied from 0.6 to 38 micro IU/ml, wherein 6.494 was the mean value. A total of 46 out of 50 patients were found to be in the normal range, and four patients had an increased value of more than 20 micro IU/ ml. These findings are approximate to our study. A study by Pan B et al. revealed that CKD patients had a rising trend in the frequency of euthyroid sick syndrome, and 69.1% of patients in CKD stage 5 had the same. Correlation analysis showed that eGFR positively correlated with T3 and FT3 [30]. A statistically significant difference (p=0.007) was recognized in our study, suggesting the reduced level of T3 with an advanced stage of CKD. Low T3 level was found more among subjects with CKD stage 5, followed by stage 4. A statistically significant difference (p=0.01) was observed with decreasing T4 levels correlating with advanced CKD stages. Lower T4 levels were most frequently found among subjects with CKD stage 5, followed by stage 4. It was reported that as the CKD stages advance, TSH levels increase, a change that was statistically significant (p=0.04). Higher TSH levels were most frequently found among subjects with CKD stage 5, followed by stage 4. All the thyroid disorders, i.e., euthyroidism, low T3 syndrome, low T4 syndrome, and hypothyroidism, were most frequently reported among subjects with stage 5 CKD, followed by stage 4, with statistically significant demographic variances. No previous studies have correlated thyroid dysfunction with different stages of CKD. The proposed mechanism of deranged thyroid in CKD may be due to the depressed hypothalamic-pituitary axis, thus reducing the expression of TSH receptors. Other hypotheses may be reduced clearance of inflammatory cytokines such as TNF-alpha and IL-1, which leads to a decrease in the level of 1 5'-deiodinase, which again leads to decreased peripheral conversion of T4 to T3, which again causes low T3 levels causing hypothyroidism [30-32].

Limitations

The main limitation was the small size of the patients with CKD. As the etiology of their CKD was not known, the correlation of the etiology of CKD with thyroid dysfunction could not be studied. Furthermore, we did not investigate thyroid dysfunction in patients on dialysis, as dialysis itself independently affects the thyroid profile.

## Conclusions

As thyroid hormone dysfunction can occur in CKD patients without any primary thyroid gland dysfunction, there may be misdiagnosis in the early stages. Our study suggests that CKD contributes to declining free thyroid hormone levels, which is more often seen as CKD progresses. Given that thyroid disorder has been associated with increased cardiovascular morbidity and mortality, CKD patients should be routinely screened for this at the primary or secondary care level. Further studies assessing the clinical importance of thyroid hormone status in patients with CKD would strengthen our understanding of the topic.
